# Supplementary material for: Angelica sinensis polysaccharide as potential protectants against recurrent spontaneous abortion: focus on autophagy regulation
Source: Front Med (Lausanne). 2025 Jan 15;12:1522503. doi: 10.3389/fmed.2025.1522503 (PMC11774876; doi:10.3389/fmed.2025.1522503)
Supplement: Supplementary material S1 — The main instruments used during the LC-MS process, along with their models/specifications and manufacturers. [file Supplementary_file_1.zip › Metabolomics sequencing data FC1.2/7.相关性分析/correlation-top20/其他图形/Exposure_Ctrl.pdf]

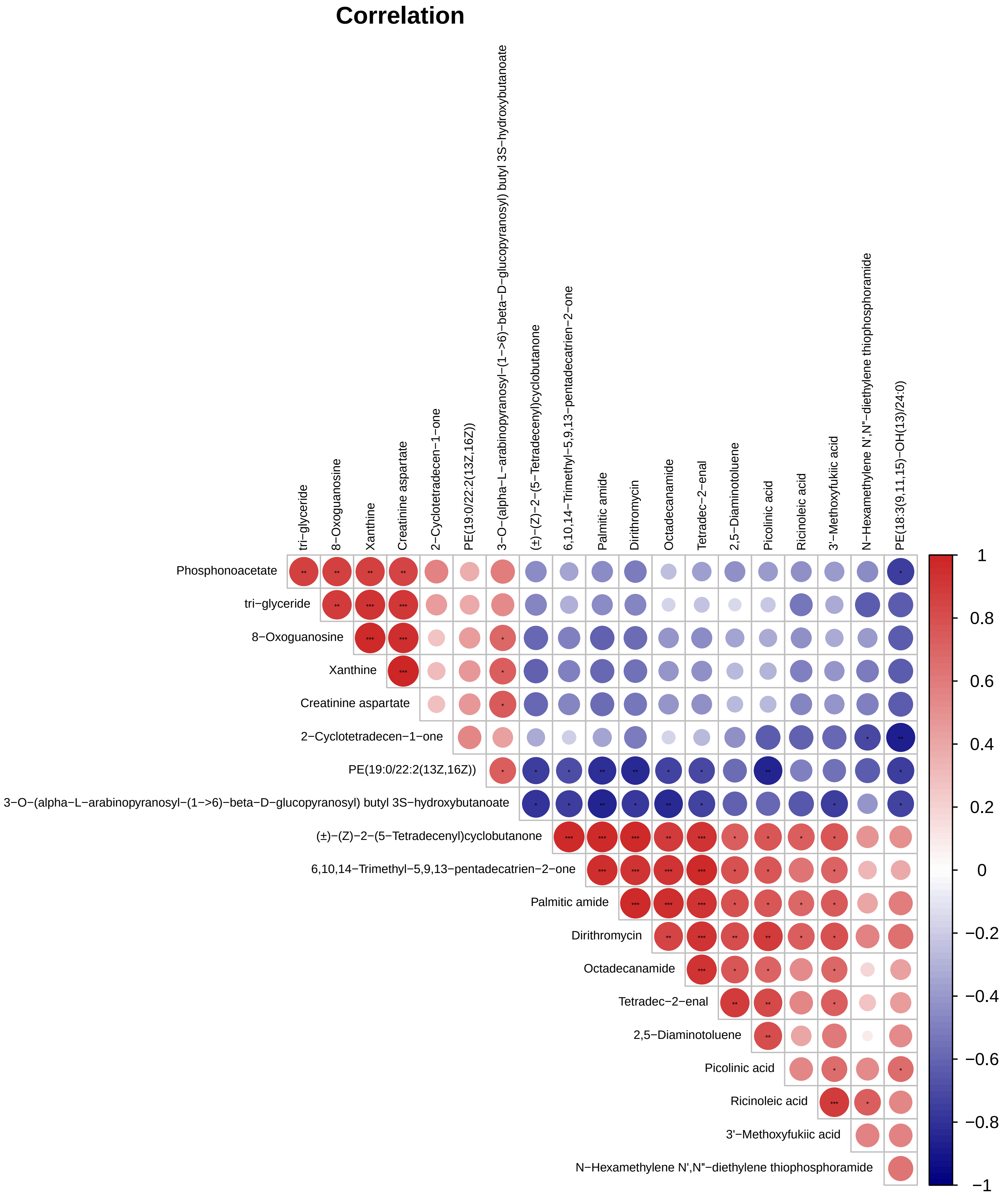
Correlation
tri−glyceride
8−Oxoguanosine
Xanthine
Creatinine aspartate
2−Cyclotetradecen−1−one
PE(19:0/22:2(13Z,16Z))
3−O−(alpha−L−arabinopyranosyl−(1−>6)−beta−D−glucopyranosyl) butyl 3S−hydroxybutanoate
(±)−(Z)−2−(5−Tetradecenyl)cyclobutanone
6,10,14−Trimethyl−5,9,13−pentadecatrien−2−one
Palmitic amide
Dirithromycin
Octadecanamide
Tetradec−2−enal
2,5−Diaminotoluene
Picolinic acid
Ricinoleic acid
3'−Methoxyfukiic acid
N−Hexamethylene N',N''−diethylene thiophosphoramide
PE(18:3(9,11,15)−OH(13)/24:0)
Phosphonoacetate
tri−glyceride
8−Oxoguanosine
Xanthine
Creatinine aspartate
2−Cyclotetradecen−1−one
PE(19:0/22:2(13Z,16Z))
3−O−(alpha−L−arabinopyranosyl−(1−>6)−beta−D−glucopyranosyl) butyl 3S−hydroxybutanoate
(±)−(Z)−2−(5−Tetradecenyl)cyclobutanone
6,10,14−Trimethyl−5,9,13−pentadecatrien−2−one
Palmitic amide
Dirithromycin
Octadecanamide
Tetradec−2−enal
2,5−Diaminotoluene
Picolinic acid
Ricinoleic acid
3'−Methoxyfukiic acid
N−Hexamethylene N',N''−diethylene thiophosphoramide
1
0.8
0.6
0.4
0.2
0
−0.2
−0.4
−0.6
−0.8
−1
